# Ergonomic risk assessment in construction: Case study Ecuador

**DOI:** 10.1016/j.heliyon.2025.e42751

**Published:** 2025-02-18

**Authors:** Oswaldo Tandazo, Victoria Jaramillo-Carrión, Eduardo Valarezo, Mercedes Sanchís-Almenara, Laura Montalbán-Domingo, Joaquín Catalá-Alís

**Affiliations:** a Departamento de Producción, Universidad Técnica Particular de Loja, Loja 1101608, Ecuador; b Departamento de Química, Universidad Técnica Particular de Loja, Loja 1101608, Ecuador; c Instituto de Biomecánica de Valencia, Universidad Politécnica de Valencia, 46022 Valencia, Spain; d School of Civil Engineering, Universitat Politècnica de València, 46022 Valencia, Spain

**Keywords:** Incorrect postures, Inadequate postures, REBA method, Ergo/IBV

## Abstract

Ergonomic risks are a global problem in construction, with health effects that have an impact on the economy. Risk assessment allows companies to identify the risks and the exposure levels to which the workers are subjected and allows to define management plans to address them. However, studies to assess risk levels through new techniques are very scarce in construction. This study aims to evaluate ergonomic risks in construction workers using Ergo/IBV (software for the assessment of ergonomic risks and design recommendations) by the observational REBA (Rapid Entire Body Assessment) method. Data were collected from 14 public sector building projects from May to November 2023. Thirty-two construction finishing activities were identified. Through the stratified method, the five most frequently executed activities were selected: interior and exterior painting, floor ceramics, wall plastering, ceiling and wall plastering. This made it possible to evaluate the ergonomic risk in 94 workers. The evaluation shows that 40 % of the workers were exposed to a high-risk level in the trunk, 2 % in the legs, 20 % in the right arm, and 22 % in the left arm. In short, the five activities adopt forced postures, with REBA scores: medium, high and very high, with high risk level being the most relevant.

## 1 Introduction

Although the construction sector has an important place in the economy of most countries, it is one of the sectors where work accidents and occupational diseases are most common [1]. Construction is set apart by competitive processes with high subcontractor involvement and an extensive supply chain. Additionally, the constantly changing work environment and tough working conditions make this industry dangerous [2]. Construction is characterized above all by intensive work activities with high exposure of employees to accidents [3]. Because of the excessive amount of activities with manual handling of loads [1], workers are constantly exposed to unsafe environments [4] and at high risk of musculoskeletal disorders (MSDs) [5] causing sick leave and economic losses, since there is a lack of prevention and protocols when starting an individual or collective task [6].

As far as residential construction workers are concerned, there is a high prevalence of MSDs due to physically demanding tasks in uncomfortable postures [7]. In this regard, studies such as Alonso, Aires and González [8], who analyzed musculoskeletal risks associated with rebar workers, Zengín and Asal [1] who evaluated the postures of employees in building construction with different ergonomic risk assessment methods, and, Domingo et al. [5] who performed a risk assessment of Filipino construction workers, have highlighted that construction activities linked with overexertion are the main causes of MSDs [8]. Musculoskeletal disorders are one of the risk factors with the highest research interest over the last two decades [[9], [10], [11]]. In order to analyze this issue, some studies have focused on recognizing construction workers' activities related precisely to overexertion as well as assessing ergonomic risk levels, which can help minimize conditions in the industry [9,12,13]. These authors concluded that there is strong evidence that many physical and psychosocial risk factors are associated with WRMSD in construction workers and that there may be great opportunity for technologies to improve the collection of musculoskeletal symptoms as part of ergonomic assessments. Therefore, analyzing risk factors in construction is essential to implement measures and improve productivity [14]. Designing the workplace based on ergonomics not only reduces risks, but also serves to favor the adaptation of workers as they return to it after an injury or illness [15].

One of the most important aspects in the selection of ergonomic risk assessment methods is to determine the points on the worker's body that feel discomfort. In this way, it is possible to decide whether to choose methods that assess the whole body or methods that assess only certain regions. Zengín and Asal [1] analyzed postures of employees in construction through the application of the Nordic musculoskeletal questionnaire. This study determined that workers working in building construction felt discomfort in their whole body. Therefore, the analysis was performed with ergonomic risk assessment methods such as REBA, RULA (Rapid Upper Limb Assessment) and OWAS (Ovako Working Analysis System) [16]. These are different postural assessment systems based on the observational method and allow a simple and quick on-site assessment at the workplace [17]. OWAS [18] is a method that consists of evaluating work positions with a set of criteria for the redesign of methods and work positions. RULA [19] identifies the risk level for developing work related musculoskeletal disorders (WRMSDs) of the upper extremities; and, REBA [20] focuses on a whole body postural analysis. The inter-method reliability for the postural loading category between OWAS and RULA is 29.2 %, and the reliability between RULA and REBA is 48.2 % [16], all designed as a field tool to assess risk exposure by assigning scores or indices based on predetermined values or postural categories [[21], [22], [23]]. The results based on overall scores and indices indicate the limits of acceptable risk exposure for workers and their levels of interventions needed to reduce risk [21].

OWAS, RULA and REBA are the three techniques that are frequently applied in industry because they allow the identification of physical strain by working posture, force and static or repetitive load [16,24]. Compared to RULA, OWAS and REBA, they generally underestimate the postural loads for the postures analyzed, regardless of industry, type of work and whether or not the body postures are in a balanced state [16]. One of the benefits of REBA is that it evaluates different parts of the body: upper limbs (arm, forearm and wrist), lower limbs, trunk and neck. It is a useful method to identify forced postures adopted by workers and develop improvement measures if necessary [25].

Observational methods are complemented by advanced techniques developed for postural assessment in highly dynamic activities [21], which record data on videos about the different activities of workers, then images are cropped for analysis by computer worksheet using software, obtaining the assessment of the working posture of workers in different activities [26]. The evaluation by RULA and REBA postural analysis indicates that workers are working above the safe limit. The highest percentage of workers present awkward postures. Therefore, workers are at moderate to high risk of musculoskeletal disorders [27].

OWAS has been applied mainly in two sectors: “Manufacturing industries” and “Care and health activities”. This method needs to be complemented with other indirect or direct methods. Moreover, whenever OWAS has been used, either individually or together with other methods, risks of musculoskeletal disorders have been detected, this being perhaps an indicator to revise the assessment parameters for overestimating the risk [28]. RULA is a survey method developed for use in ergonomic investigations of workplaces where work-related upper extremity disorders are reported. This tool requires no special equipment to provide a rapid assessment of neck, trunk and upper extremity postures along with muscle function and external loads experienced by the body. A coding system is used to generate a list of actions that indicates the level of intervention needed to reduce the risks of injury due to physical loading of the operator [19].

REBA has been developed to meet the perceived need for a field tool for practitioners, specifically designed to be sensitive to the type of unpredictable work postures encountered in industry. Five risk levels are considered in the REBA score, in turn, a certain action level for ergonomic intervention (need to take action to reduce the risk): action level 0, corrective action including further assessment is not necessary; action level 1, corrective action including further assessment may be necessary; action level 2, corrective action including further assessment is necessary; action level 3, corrective action including further assessment is necessary soon; and action level 4, corrective action including further assessment is now necessary, as shown in Table 1 [20].

**Table 1:** REBA score, risk level and action level.

REBA Score	Level of Risk	Level of Action
1	Negligible	0. Not needed
2–3	Low	1. May be necessary
4–7	Medium	2. Needed
8–10	High	3. Needed soon
11–15	Very high	4. Needed NOW

Some studies show that the most stressful tasks observed were sawing, positioning and hammering for carpentry-related workers, positioning and tying of iron rods for iron workers, brick laying for masonry, and manual handling of steel structures in confined spaces for scaffolding workers [1]. Studies such as risk assessment of Filipino construction workers show that tasks such as using shovels as hand tools, painting with a roller, demolition from a higher level, floor polishing among the main ones, carry higher level of risk Domingo [5]. Most of these activities the trunk condition of the construction worker is in a non-neutral posture when executing the tasks and exposed to MSDs [29]. At this point, the quantitative assessment of risks by ergonomists is still carried out with pencil and paper [13], an alternative for ergonomic assessment is the use of mobile device applications. Several of the systematic observational based assessment methods have increased in use by certified ergonomics practitioners, those being: RULA, REBA, Psychophysical Upper Extremity Data, Strain Index, and American Conference of Government Industrial Hygienists (ACGIH) Threshold Limit Value (TLV) for Hand Activity Level [12,13].

The use of the REBA method has increased in the last decade, probably due to the digitalization of knowledge. It is almost always applied in combination with other methods, and its use can be a positive indicator of company sustainability. However, to date in Ecuador no ergonomic risk assessment in construction has been carried out using the RULA method. For this reason, the authors propose to carry out this study with the aim to evaluate the ergonomic risks to which workers in the construction sector in Ecuador are exposed, especially those workers assigned to finishing activities in buildings, in order to determine the level of risk and the level of action required. Finishing activities in buildings are relevant because of the high physical demands due to posture and repetition and because of the significant incidence of injuries and diseases that are generated in this group of workers. To assess ergonomic risks, the REBA method was used to collect data on site, as well as the Ergo/IBV software (Instituto de Biomecánica, Valencia, Spain) and the standard numerical evaluation scale proposed by Chiasson et al., in 2012 for processing and obtaining results [30].

## 2 Methodology

**Fig. 1:**
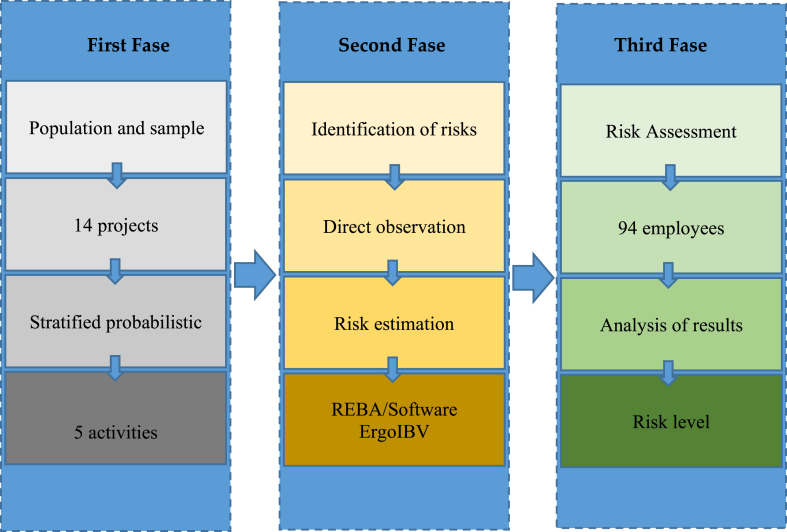
General scheme of the research method.

The main objective of this study is to evaluate the ergonomic risk factors in workers of the construction sector in Ecuador. For this purpose, a methodology composed of three phases was used. Fig. 1 shows the general scheme of the research method according to the objective of the study, to evaluate the ergonomic risk factors in construction workers in building finishing activities.

In the first phase, construction projects that contemplated the execution of finishing activities were characterized and selected. For this purpose, the public contracting construction projects in a state of execution in the city of Loja-Ecuador, from May to November 2019, were characterized. The characterization allowed prioritizing the projects that contemplated the performance of construction finishing activities in their budgets in the aforementioned period. Fourteen projects with different activities, were characterized. The stratified probabilistic method was applied to select the activities to be studied and the repetitiveness index of the task was taken into account as a criterion for the incidence of MSDs, injuries and/or illnesses. Based on the application of this index, five activities were selected to be evaluated.

In the second phase, a questionnaire was defined to identify, based on direct observation, the main risk factors in the five selected activities. This observation was carried out by a senior evaluator and a junior evaluator, both with specific training in occupational risk prevention and knowledge of applied ergonomics. The questionnaire used was the ErgoCheck. This questionnaire contains 12 basic questions to identify situations that may involve ergonomic risks. In Level I all the items of the questionnaire must be reviewed, and the relevant box must be checked when this situation exists in the workplace or task under analysis. Once Level I has been finished (risks identification, Fig. 1), the user can complete the tabs of Level II, where the data collection was based on the direct observation of the workers in the workplace or task (observation direct). Once Level II has been completed, it is possible to access the results tab, which contains information about risk factors, recommendations to help to correct the problem detected and recommended module. As a result of the ErgoCheck questionnaire the software recommends the most adequate modules to estimate ergonomic risk with REBA/ErgoIBV.

Although physical exposure to potential musculoskeletal injury risks has been assessed using a variety of methods, such as: paper-and-pencil based observation, videotaping and computer-assisted analysis, direct or instrumental techniques and, self-assessment approaches [17], the direct observation method is the most widely used because it is inexpensive to apply, easy to use, flexible and does not interfere with the performance of the tasks executed by the workers [31]. Despite the advantages of the existing observation methods to identify ergonomic hazards, these are rarely used on construction sites because they are time-consuming, and subject to observer bias [7]. With training, observers can achieve consistent results in clearly visible body postures and work activities [32]. Observational assessment methods seem to be better suited to the needs of practitioners than direct measurement methods.

To strengthen the analysis of the results, photographic and video records were taken, which will later allow for estimating the risks using REBA and the ErgoIBV software. For this, initially based on the image (photograph) analyzed, the avatar corresponding to the trunk, neck and legs is marked in the software, as well as information on the characteristics of whether it includes rotation or inclination of the body segments (section A). Then, information is entered for the arm, forearm, wrist, both right and left, as well as characteristics that include abduction, rotation or elevation, as well as rotation and deviation in the wrists (Section B). Finally, information on strength, grip and activity is entered and the results are disaggregated by group A and B, as well as by body parts, and the software automatically calculates the REBA score associated with a risk level.

In the third phase, the REBA ergonomic evaluation method of postural analysis was applied. The usefulness of the REBA method lies in the fact that it provides a subjective forecast of the potential risks to which the worker is exposed. Therefore, the risk was evaluated based on the posture of the trunk, arm and hands through the Ergo/IBV software. Which allowed us to calculate the level of risk that the worker has to suffer musculoskeletal ailments of work origin [33]. Postural analysis can be an excellent technique to assess work activities. The risk of MSDs associated with recorded postures, in the context of a complete ergonomic evaluation of the workplace, can be an important factor in implementing change, so the availability of task-sensitive field techniques is of great help to the ergonomics specialist [34].

## 3 Results and discussion of results

Fourteen public procurement projects for building maintenance were executed. In each project, 32 construction finishing activities were found. Table 2 shows the 32 construction finishing activities and the number of times they are executed in each public contracting project for building maintenance. The activities from AC1 to AC32 are all the activities identified in the 14 projects in which the study was carried out. Of these 32 activities, the 5 with the greatest repetitiveness (>7 times) were stratified and considered for the study, which are from AC1 to AC5. The activities from AC6 to AC32 are executed between 1 and 6 times within all contractual building works projects. However, in Table 2 all the activities identified in the projects are shown, so that their nature is known, and this information will serve as a reference for later studies.

**Table 2:** Results of construction finishing activities within the 14 public procurement projects.

AC	Description	Project														NTE	NWE
		1	2	3	4	5	6	7	8	9	10	11	12	13	14		
AC1	Interior and exterior painting	**✓**	**✓**		**✓**	**✓**		**✓**	**✓**	**✓**	**✓**	**✓**	**✓**	**✓**	**✓**	12	20
AC2	Ceramic floor tile installation	**✓**	**✓**	**✓**			**✓**	**✓**	**✓**	**✓**	**✓**		**✓**	**✓**	**✓**	11	20
AC3	Wall plastering		**✓**		**✓**		**✓**	**✓**	**✓**	**✓**	**✓**	**✓**	**✓**		**✓**	10	20
AC4	Ceiling installation	**✓**	**✓**		**✓**	**✓**	**✓**	**✓**	**✓**	**✓**	**✓**				**✓**	10	20
AC5	Wall and slab plastering		**✓**		**✓**			**✓**	**✓**	**✓**	**✓**	**✓**				7	14
AC6	Ceramic wall tile installation		**✓**				**✓**	**✓**	**✓**	**✓**			**✓**			6	–
AC7	Door installation		**✓**		**✓**	**✓**	**✓**		**✓**	**✓**						6	–
AC8	Window installation		**✓**		**✓**		**✓**	**✓**	**✓**	**✓**						6	–
AC9	Installation of sanitary parts				**✓**		**✓**	**✓**	**✓**	**✓**			**✓**			6	–
AC10	Glass installation		**✓**						**✓**	**✓**	**✓**				**✓**	5	–
AC11	Chrome faucet installation	**✓**	**✓**					**✓**							**✓**	4	–
AC12	Window protection		**✓**		**✓**				**✓**	**✓**						4	–
AC13	Base board				**✓**				**✓**	**✓**					**✓**	4	–
AC14	Wooden floor					**✓**	**✓**		**✓**						**✓**	4	–
AC15	Autocoband coating on facade		**✓**	**✓**			**✓**									3	–
AC16	Porcelain tile installation		**✓**		**✓**										**✓**	3	–
AC17	Kitchen furniture	**✓**	**✓**													2	–
AC18	Night and day type blind				**✓**										**✓**	2	–
AC19	High melamine furniture				**✓**		**✓**									2	–
AC20	Aluminum partition				**✓**										**✓**	2	–
AC21	Granite countertop						**✓**	**✓**								2	–
AC22	Ceramic tile countertop		**✓**													1	–
AC23	Stuccoed interior and exterior walls														**✓**	1	–
AC24	Aluminum partition installation						**✓**									1	–
AC25	Sink installation		**✓**													1	–
AC26	Installation of odor hood		**✓**													1	–
AC27	Coated with cutting edges		**✓**													1	–
AC28	Cubilar curtain				**✓**											1	–
AC29	Floor puttying				**✓**											1	–
AC30	Wood lacquering				**✓**											1	–
AC31	Decorative vinyl for wall				**✓**											1	–
AC32	Hand wash basin cabinet						**✓**									1	–
														**Total workers**			**94**

AC: Activity; NTE: Number of times executed; NWE: Number of workers evaluated.

The activity AC1: Interior and exterior painting is executed 12 times, AC2: Laying of ceramic tiles on the floor is executed 11 times, AC3: Plastering of walls, and AC4: Laying of ceiling are executed 10 times and AC5: Plastering of walls and slab is executed 7 times. The number of workers evaluated is also shown, which is 94, with respect to the 5 activities that are executed more times considered as the research strata. These activities concentrate the interest in the study as they are the activities with a greater presence in the projects.

**Fig. 2:**
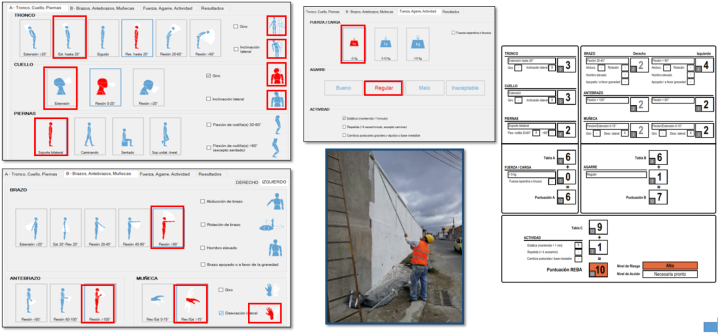
Scoring body segments of the body.

For illustrative purposes, Fig. 2 shows an example of how the information is entered into the Ergo/IBV software according to the body segments and the finishing activity of the construction carried out.

Table 3 shows the activities, as well as the description of tasks 1 and 2. Also the relationship with the position of construction workers such as bricklayer, laborer, painter and coating installer. Also, regarding the risk assessment, it shows the number of workers evaluated for each task, the REBA score obtained (mode), the level of risk of musculoskeletal injury and level of necessary action. The results show mode of value of risk level (score 5) in paint placement, a high risk level (score 10) in ceramic placement and a very high risk level (score 11) in handling cement bags, with levels of actions necessary, necessary soon and necessary now respectively [20].

**Table 3:** Task risk level results by construction finishing activity.

AC	Activity description						Risk Assessment			
	Task		Construction workers				NWE	REBA score	Risk Level	Level of action
			Mason	Laborer	Painter	Siding installer				
AC1	1	Paint placement			**✓**		10	5	Medium	Needed
	2	Manual handling of loads (gallon of paint)		**✓**			10	10	High	Needed soon
AC2	1	Ceramic floor tile installation	**✓**				10	10	High	Needed soon
	2	Manual handling of loads (ceramic box)		**✓**			10	10	High	Needed soon
AC3	1	Wall plastering	**✓**				10	9	High	Needed soon
	2	Manual handling of loads (cement sack)		**✓**			10	11	Very High	Needed NOW
AC4	1	Ceiling installation				**✓**	10	9	High	Needed soon
	2	Ceiling filling	**✓**				10	9	High	Needed soon
AC5	1	Wall plastering	**✓**				7	9	High	Needed soon
	2	Manual handling of loads (filling bag)		**✓**			7	10	High	Needed soon

The REBA method for each posture analyzed, also allows to obtain results (mode) for task 1 and task 2 (Table 4) by the position of the different body segments, comprising trunk (1–4), neck (1–2), legs (1–2), arms (1–4), forearms and wrists (1–2), including the strength score, type of grip and muscle activity involved in that posture.

**Table 4:** Results of tasks 1 by body zones.

AC	T (5)	N (3)	L (4)	RA (6)	LA (6)	RLA (2)	LLA (2)	RW (3)	LW (3)	Description of the score
**Tasks 1**										
AC1	3	3	1	2	1	2	2	2	2	A **high level** of risk is obtained in the posture of the worker's **trunk** and **neck**, in addition to the postural risk in the **lower arms**.
AC2	4	2	3	2	2	2	2	2	2	A **high level** of risk is obtained in the posture of the worker's **trunk** and **legs**, in addition to the postural risk in the **lower arms**.
AC3	4	2	2	2	2	2	2	2	1	A **high level** of risk is obtained in the posture with respect to the worker's trunk, in addition to the postural risk in the forearms.
AC4	3	2	1	4	4	2	2	3	3	A **high level** of risk is obtained in the **trunk**, **lower arms** and **wrists** of the worker,
AC5	3	2	1	5	2	2	2	3	0	A **high level** of risk is obtained in the **trunk**, **right arm**, **lower arms** and **right wrist** of the worker.
**Tasks 2**										
AC1	2	2	1	2	5	2	2	3	2	A **high level** of risk is obtained in the posture of the worker's **left arm**, in addition to the postural risk in the **lower arms** and **right wrist**.
AC2	4	2	1	2	2	2	2	3	3	A **high level** of risk is obtained in the posture with respect to the worker's **trunk**, in addition to the postural risk in the **wrists**.
AC3	4	2	3	2	2	2	2	3	3	A **high level** of risk is obtained in the posture with respect to the worker's **trunk**, in addition to the postural risk in the **lower arms** and **wrists**.
AC4	3	2	1	5	1	2	2	3	1	A **high level** of risk is obtained in the posture of the worker's **trunk, right arm**, **lower arms** and **right wrist**.
AC5	4	2	1	2	2	2	2	3	2	A **high level** of risk is obtained in the posture with respect to the **trunk** and **lower arms** and **right wrist** of the worker.

T: Trunk; N: Neck; L: Legs; RA: Right arm; LA: Left arm; RLA: Right lower arm; LLA: Left lower arm; RW: Right wrist; LW: Left wrist.

The study allowed the evaluation of the risk factors with a final REBA score that represents the level of risk of musculoskeletal injury of the different working postures in the construction finishing activities in the two tasks analyzed and that implies a certain level of action to reduce the risk. It was obtained that 40 % of the total workers (sample) present a high-risk level in the trunk, 2 % present a high-risk level in the legs, 20 % a high-risk level in the right arm and 22 % in the left arm. Umer et al., in 2018 found that low back pain was the most common work-related musculoskeletal disorder (MSD) among workers (51 %), while other commonly affected body parts were knee (37 %), shoulder (32 %) and wrist (30 %) [35]. It also revealed that most construction workers routinely worked in an awkward operating posture and were affected by alterations in musculoskeletal manifestations such as low back, neck and wrist pain. Often these awkward operating postures include raising the arms and twisting the trunk to reach materials. According to the REBA score results obtained by body zones we can corroborate what was indicated by the European Foundation for the Improvement of Living and Working Conditions, who determined that 43 % of European workers have problems in the back, followed by muscular pain in the neck or in the upper part with respect to the extremities with 42 % and 29 % in lower limbs or legs [36].

In the two tasks of each of the five construction finishing activities evaluated, forced postures are adopted, the construction workers must remain standing and work at floor level during the workday, the final REBA score determined is within the parameters: medium, high and very high, being the most relevant the high risk level, for this reason it is evident the alert that the performance of these tasks would trigger musculoskeletal disorders in construction workers. Proper work and rest schedule, application of some work techniques and use of some ergonomically designed equipment can decrease MSDs at work and improve the health of construction workers in unorganized sectors (Chatterjee & Sahu, 2018). Low back pain is the most common work-related MSD followed by shoulder, neck and knee pain in construction workers [[37], [38], [39], [40], [41], [42], [43]]. Wrist and hand pain among construction workers was also commonly reported in many studies [38,40,41], these assertions in these studies are similar to those found in ours as stated above. These findings highlight the enormous negative impacts of MSDs on the economy and productivity in the construction industry [44].

Our study provides relevant information to the scientific community, corroborating that the prevalence of work-related MSDs is very high among construction workers worldwide [45], Ecuador is no exception. Goldsheyder et al. states that approximately 77 % of United States of America (USA) construction workers reported at least one musculoskeletal symptom in the last 12 months [41]. It has been estimated over 400 USD million in workers' compensation incurred annually due to work-related MSDs in the USA construction industry [46]. Low back, neck, and upper extremities are the most commonly affected body parts [35]. In addition to physical affection, work-related MSDs can generate huge financial burdens and absenteeism in the construction industry [47], so it becomes important to assess the risks in order to manage them properly.

## 4 Conclusions

The construction industry is one of the most dangerous sectors, especially due to the uncomfortable working postures and a priori that workers perform in different activities. Therefore, it is important to permanently evaluate the risk factors present in the construction industry in order to periodically quantify the levels and be able to intervene effectively. It is important to apply technological tools that allow optimizing time in the collection and analysis of data within these applications is Ergo/IBV, a tool that allowed evaluating the risks in less time as opposed to other studies that take longer in the evaluation.

The results show a high level of risk in some tasks, especially in the neck and trunk, but also in the arms and wrists. REBA divides the body into segments that are coded individually, in relation to the planes of movement, also providing a scoring system for muscle activity caused by static, dynamic, unstable or rapidly changing postures. The construction projects require a lot of time for the analysis of all its activities, due to its duration in terms of execution, being vital to stratify and study the most risky, in Ecuador are characterized by high staff turnover which makes difficult the management of ergonomic risks regarding their prevention, however, conducting studies with the use of applications such as Ergo/IBV software decreases analysis time allowing a prompt response to the level of risk that can produce an injury or illness in workers.

An extension of the study is recommended in order to establish preventive measures for each of the tasks in the activities, apply them and with the results obtained determine whether or not there is a decrease and control of existing ergonomic risks. Future studies should be followed up to continue examining ergonomic exposure during construction work to prevent ergonomic injuries and musculoskeletal disorders for workers. Future studies are warranted to quantify the incidence of work-related MSDs in different construction projects. For manual load handling tasks, an additional study is recommended to complement the results obtained from the level of ergonomic risk present, with the use of observational methods through quantitative analysis tools, as appropriate. With this study on the evaluation of ergonomic risks in construction, the authors leave the doors open for future research on musculoskeletal discomfort, workstation design, ergonomic design of tools and their direct relationship with the improvement of work environments with respect to the prevention of occupational hazards.

## CRediT authorship contribution statement

**Oswaldo Tandazo:** Writing – review & editing, Project administration, Investigation, Formal analysis, Conceptualization. **Victoria Jaramillo-Carrión:** Methodology, Investigation, Formal analysis. **Eduardo Valarezo:** Writing – original draft, Validation, Data curation. **Mercedes Sanchís-Almenara:** Writing – review & editing, Validation, Data curation. **Laura Montalbán-Domingo:** Writing – review & editing, Supervision, Investigation, Conceptualization. **Joaquín Catalá-Alís:** Visualization, Validation, Supervision.

## Data availability statement

Data will be made available on request. For requesting data, please write to the corresponding author.

## Declaration of competing interest

The authors declare that they have no known competing financial interests or personal relationships that could have appeared to influence the work reported in this paper.
